# Inflammatory and lipotoxicity mechanisms in obesity related CKD

**DOI:** 10.3389/fneph.2025.1684004

**Published:** 2026-01-14

**Authors:** Jorge Rico-Fontalvo, Maria Raad-Sarabia, Juan Montejo Hernández, Tomas Rodríguez Yánez, Lacides Rafael Caparroso Ramos, Paula Parra Sánchez, Ana Alexandra Ovalle Gomez, Javier Jimenez Quintero, Rodrido Daza-Arnedo

**Affiliations:** 1 Asociación Colombiana de Nefrología e HTA (ASOCOLNEF), Bogotá, Colombia; 2 Facultad de Medicina, Departamento de Nefrología, Universidad Simón Bolívar, Barranquilla, Colombia; 3 Departamento Médico, Nephromedicall Institución Prestadora de Salud (IPS), Medellín, Colombia; 4 Facultad de Medicina, Departamento de Medicina Interna, Universidad del Sinú, Cartagena, Colombia; 5 Unidad de Cuidado crítico, Clínica Gestión Salud, Cartagena, Colombia; 6 Departamento de Medicina Interna, Hospital Universitario Clínica San Rafael, Bogotá, Colombia; 7 Departamento de medicina interna, Fundación Universitaria Juan N Corpas, Bogotá, Colombia; 8 Facultad de Medicina, Universidad Militar Nueva Granada, Bogotá, Colombia

**Keywords:** inflammation, lipotoxicity, cytokines, obesity, chronic kidney disease

## Abstract

Obesity has been a systemic disease that has been underrecognized for years. Obesity-related chronic kidney disease (Ob-CKD) is a multifaceted disorder that affects patients with CKD to varying degrees. Among the structural changes associated with obesity, obesity-related glomerulopathy (ORG) stands out (glomerular hypertrophy, podocytopathy, mesangial matrix expansion, focal segmental glomerulosclerosis, tubulointerstitial fibrosis, vascular lesions, and tubular atrophy) associated with other kidney diseases. There are direct and indirect mechanisms that affect the kidneys of obese patients. Among the direct mechanisms, several effects may occur: hyperfiltration, activation of the renin-angiotensin-aldosterone system (RAAS), inflammation, lipotoxicity, and neurohormonal activation. This is a narrative review that will detail the inflammatory and lipotoxicity mechanisms involved in the genesis of Ob-CKD.

## 1 Introduction

Obesity is a major determinant of premature death and a significant cardiovascular risk factor that contributes to the development of a broad spectrum of chronic non-communicable cardiovascular diseases (1). In this context, it is a frequent cause of early mortality shared by several chronic non-communicable conditions, including diabetes mellitus (DM), coronary artery disease (CAD), hypertension (HTN), cerebrovascular disease (CVD), various types of cancer, and chronic kidney disease (CKD) (1, 2). CKD, in particular, is associated with poorer clinical outcomes, a marked decline in quality of life, and serious systemic complications—such as cardiovascular disease, mineral and bone metabolism disorders, anemia, acid-base imbalances, and fluid overload—resulting in a significant healthcare burden (3–5).

According to recent data from the World Health Organization (WHO), in 2022 approximately 43% of adults worldwide were overweight, and 16% were living with obesity—equivalent to nearly 890 million individuals (6). Projections indicate that by 2035, over 1.5 billion adults may be affected by obesity, with the greatest burden expected in low- and middle-income countries (6). This persistent trend is strongly associated with the rising incidence of non-communicable diseases, including cardiovascular disease, type 2 diabetes, certain malignancies, and chronic respiratory conditions, all contributing to increased morbidity and premature mortality.

The global surge in obesity prevalence has been paralleled by a rise in cases of obesity-related glomerulopathy (ORG). This condition is morphologically defined by glomerulomegaly and, frequently, by focal segmental glomerulosclerosis (FSGS) lesions, predominantly of the perihilar subtype (7, 8). These histopathological findings are often identified in renal biopsies of obese individuals, even in the absence of comorbidities such as diabetes mellitus or hypertension (7, 8). The diagnosis of ORG is established by excluding other causes of kidney disease through thorough clinical and histological evaluation in individuals with a body mass index (BMI) greater than 30 kg/m² (7).

The precise mechanisms by which obesity contributes to the onset and progression of CKD remain incompletely understood. Nonetheless, several pathophysiological pathways have been proposed, generally categorized into direct and indirect mechanisms (7, 9). Direct mechanisms include glomerular hyperfiltration, inflammatory activation, oxidative stress, hormonal dysregulation, and expansion of perirenal and renal sinus adipose tissue (10–12). Indirect mechanisms act through the increased risk of developing metabolic syndrome, type 2 diabetes mellitus, and hypertension—conditions that collectively accelerate renal injury (11, 13).

**Figure 1:**
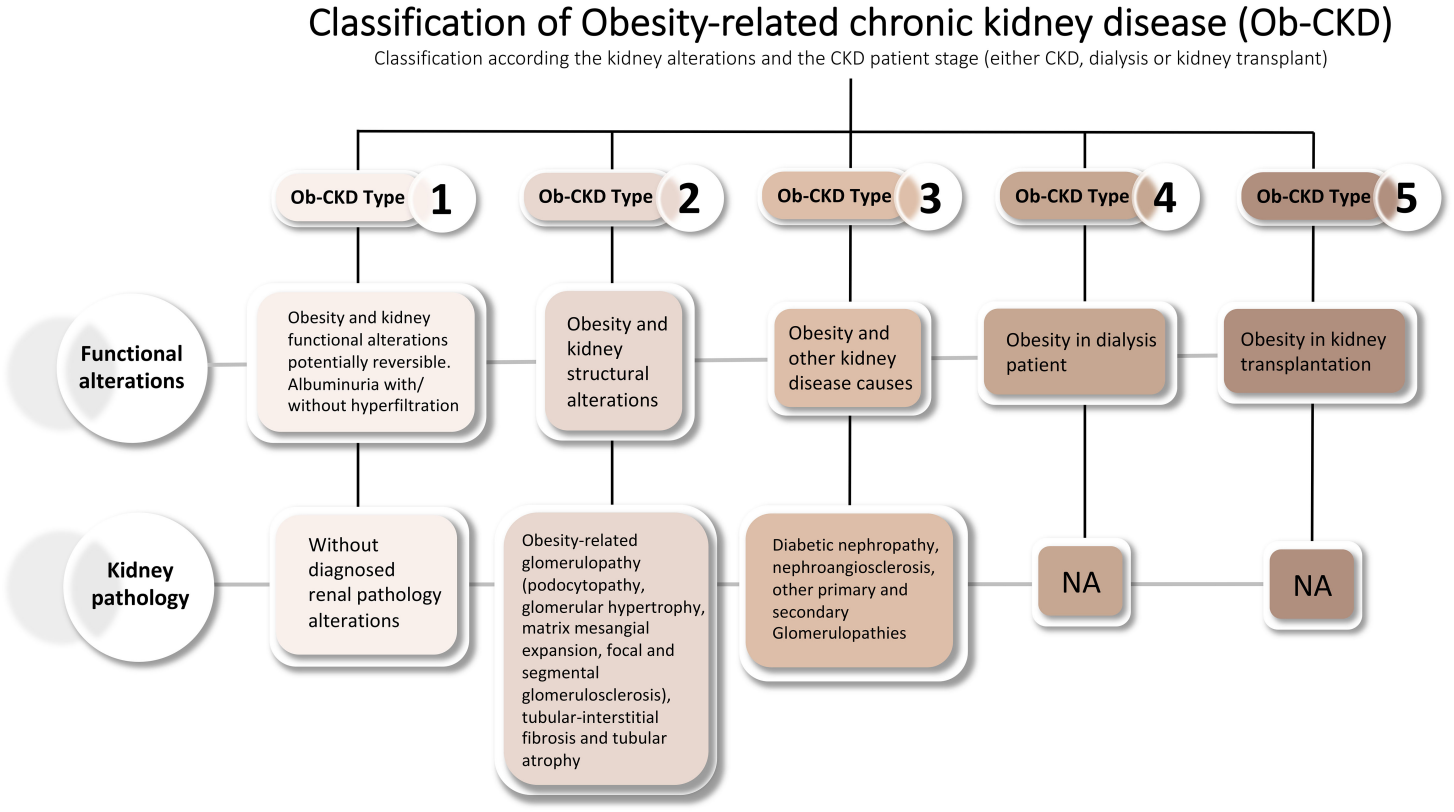
Classification Ob-CKD. Ob-CKD, obesity-related chronic kidney disease; CKD, chronic kidney disease; NA, not applicable. Adapted from Rico-Fontalvo et al. (2).

In light of these considerations, and based on the wide clinical spectrum of CKD—including patients on dialysis or those with kidney transplants—a recent consensus has proposed a novel classification system for Obesity-related CKD (Ob-CKD), categorizing the condition into five distinct subtypes, as shown in **Figure 1** (2, 14).

Recently, increasing attention has been directed toward the role of chronic inflammation and lipotoxicity as central mediators in the pathophysiology of Ob-CKD. Within this context, the present review aims to examine the pathophysiological mechanisms underlying the development of Ob-CKD, with a particular focus on the contribution of chronic inflammation and lipotoxicity.

## 2 Methods

A narrative review was conducted to synthesize relevant elements and summarize the available evidence on the role of lipotoxicity and inflammation in the course of obesity-related kidney disease (Obesity-related glomerulopathy).

A literature search was conducted in PubMed starting January 1, 2000, with no date or country restrictions. However, only full-text articles published in English were included. The following combination of MeSH terms and keywords was used in PubMed: “Inflammation” OR “lipotoxicity” OR “cytokines” OR “obesity” OR “chronic kidney disease” OR “enfermedad renal cronica asocia a obesidad” OR “of obesity-related glomerulopathy.” This search yielded 417,148 results in PubMed. After the review, 54 articles were included based on relevance.

## 3 Obesity and kidney disease: histopathological and pathophysiological changes

Obesity is a condition characterized by excessive fat accumulation accompanied by adipocyte hyperplasia and hypertrophy. It has a multifactorial etiology and is phenotypically defined by a body mass index (BMI) greater than 30 kg/m² (15). In recent decades, we have witnessed an exponential rise in its prevalence, with obesity now recognized as a leading cause of chronic kidney disease (CKD) after diabetes mellitus, as well as a frequent contributor to its development (15).

Obesity adversely affects renal function through a complex network of both direct and indirect pathophysiological mechanisms, all of which ultimately converge in structural deterioration of the renal parenchyma (16). Direct mechanisms are primarily linked to excessive adipose tissue accumulation and include hemodynamic alterations, hormonal dysfunction, and localized inflammatory responses. In contrast, indirect mechanisms are mediated by comorbidities closely associated with obesity—such as hypertension, type 2 diabetes mellitus, and atherosclerosis—which collectively accelerate the progression of CKD (16–19).

### 3.1 Lipids and glomerulus

Renal damage induced by obesity is both structural and functional in nature (20). Structurally, glomerulomegaly is identified as the primary histopathological alteration associated with obesity-related glomerulopathy (ORG), representing a key morphological finding for its diagnosis (13). Glomerular hypertrophy constitutes an early and critical lesion that precipitates podocyte dysfunction and effacement, promoting a localized inflammatory response with the release of proinflammatory cytokines (8). This cascade ultimately leads to focal segmental glomerulosclerosis, predominantly affecting the perihilar region of the glomerulus (7, 20).

Additionally, the activation of profibrotic signaling pathways facilitates the accumulation of extracellular matrix, contributing to glomerular basement membrane thickening, glomerulosclerosis, and tubulointerstitial fibrosis (7).

As the disease progresses, the expansion of the glomerular surface area exceeds the podocytes’ capacity to maintain adequate coverage, leading to their dysfunction and subsequent loss of filtration barrier integrity, along with overload of the remaining cells. This process ultimately results in renal injury characterized by glomerular hyperfiltration and proteinuria (7, 8, 20).

However, not all individuals with obesity or an elevated body mass index (BMI) develop CKD, suggesting that an increased BMI alone is insufficient to trigger the disease. Additional metabolic disturbances appear to be required for its onset and progression (7).

Another common histological alteration associated with ORG is focal segmental glomerulosclerosis (FSGS), which is defined by the presence of segmental sclerotic lesions affecting a subset of glomeruli, with partial collapse of the involved glomerular capillaries (21). Among the morphological variants of FSGS, the perihilar subtype is the most frequently observed in the context of ORG, likely as a consequence of associated glomerulomegaly—a characteristic feature of chronic glomerular hypertension and hyperfiltration induced by obesity (22).

In addition, hypertrophied podocytes or podocytes displaying vacuolar changes may be observed, along with proliferating parietal epithelial cells lining sclerotic areas, further contributing to the altered glomerular architecture characteristic of FSGS lesions in ORG (22).

### 3.2 Renal blood vessels, renal tubule and lipids effects

Regarding the involvement of renal blood vessels, characteristic morphological changes have been identified in the small renal arteries. These include dilation of the glomerular arterioles and peripheral capillaries adjacent to the glomerular vascular pole, which have been attributed to increased intraglomerular flow as well as elevated local perfusion and plasma pressure (22). Although findings such as arterial intimal thickening and arteriolar hyalinosis have been documented in some cases, no specific histopathological lesions have yet been described in arteries, arterioles, peritubular capillaries, or renal veins that are exclusive to ORG.

On the functional level, obesity can induce direct glomerular damage through hemodynamic alterations, primarily mediated by afferent arteriolar vasodilation and increased proximal tubular sodium reabsorption. These changes lead to sustained glomerular hyperfiltration, which in advanced stages clinically manifests as proteinuria—typically in the subnephrotic range—constituting a key clinical feature in patients with Ob-CKD (7, 23).

Lipotoxicity at the renal level has been associated with both structural and functional alterations in various renal cell types, including mesangial cells, podocytes, and proximal tubular epithelial cells (24). In podocytes, lipotoxicity impairs insulin signaling, a pathway essential for maintaining their structural integrity and cellular viability. This disruption promotes podocyte apoptosis and triggers a compensatory hypertrophic response by the remaining podocytes, contributing to the progressive deterioration of the glomerular filtration barrier (20).

Among the pathophysiological mechanisms involved in Ob-CKD, hemodynamic and metabolic overload of the kidney induced by obesity stands out, promoting glomerular hyperfiltration, increased proximal tubular sodium reabsorption, thickening of the glomerular basement membrane, and ultimately the development of glomerulosclerosis (25). It is worth noting that this pattern of hyperfiltration-related injury is also observed in other conditions such as arterial hypertension, further supporting its role as a common mechanism in the progressive deterioration of renal function. **Table 1** summarizes the principal histopathological and physiological changes that characterize Ob-CKD.

**Table 1:** Different pathways of nephrotoxicity in obesity.

Pathway or mechanism	Description	Effect on the kidney
Lipotoxicity	Excess free fatty acids and triglycerides are deposited in podocytes, mesangial cells, and proximal tubular cells.	Cellular dysfunction, endoplasmic reticulum stress, and apoptosis
Mitochondrial dysfunction	Lipid overload impairs mitochondrial function and increases the production of reactive oxygen species	Oxidative damage, inflammation, and cell death.
Activation of inflammatory pathways (NF-кВ, JAK/STAT, MAPK)	The lipotoxic environment induces the expression of pro-inflammatory cytokines such as TNF-α, IL-6, and MCP-1.	Chronic renal inflammation, fibrosis, and progression of CKD.
Impaired insulin signaling	Excess lipids interfere with insulin signaling pathways in podocytes and other renal cells.	Podocyte apoptosis and disruption of the glomerular filtration barrier.
Dysfunctional adipokine secretion	Increased leptin levels, reduced adiponectin, and elevated resistin concentrations are observed.	Promotion of profibrotic effects, glomerular hypertrophy, and glomerulosclerosis.
Perirenal Adipose Tissue- Induced Inflammation	Perirenal adipose tissue secretes inflammatory mediators and exerts compressive effects on renal vasculature.	Intrarenal hypertension, decreased GFR, and proteinuria.

NF-kB, Nuclear factor kappa B; TNF-a, Tumor necrosis factor alpha; IL-6, Interleukin 6; MCP-1, Monocyte chemoattractant protein-1; CKD, Chronic kidney disease; GFR, Glomerular filtration rate.

## 4 Inflammation, obesity, and chronic kidney disease

It has been established that obesity is a disease characterized by a chronic proinflammatory state with multiple associated comorbidities (26). In addition to serving as an energy reservoir, adipose tissue functions as an endocrine organ and is infiltrated by various cellular populations, including macrophages and other immune-active cells such as T and B lymphocytes and dendritic cells (26). In fact, most of the total body fat is functionally considered part of the endocrine organ system. The dysfunction of this tissue, in response to sustained positive caloric balance in genetically susceptible individuals, contributes both directly and indirectly to the development of cardiovascular and metabolic diseases, including CKD. This dysfunction, known as “adiposopathy,” is supported by three main pathophysiological mechanisms: hemodynamic alterations, metabolic disruptions, and a chronic inflammatory response—hallmarks of obesity (27).

The persistent inflammatory state that characterizes obesity leads to maladaptive mechanisms that generate oxidative stress and cellular damage, affecting peripheral tissues beyond adipose tissue, including the kidneys. In this context, white adipose tissue is a complex and highly functional endocrine organ, which includes various cellular populations such as adipocytes, endothelial cells, preadipocytes, leukocytes, macrophages, monocytes, and fibroblasts (28). These cells mediate the release of various inflammatory processes through the endogenous production of cytokines and nephrotoxic adipose tissue-derived mediators, such as tumor necrosis factor-alpha (TNF-α), leptin, interleukin-6 (IL-6), monocyte chemoattractant protein-1 (MCP-1), resistin, visfatin, and plasminogen activator inhibitor-1 (PAI-1), all of which exert deleterious effects on the kidney (1).

The activation of inflammatory pathways such as NF-κB, the increase in reactive oxygen species (ROS), and the involvement of protein kinase cascades—including the mitogen-activated protein kinase (MAPK) pathway and JAK-STAT–mediated signaling—play a central role in the pathophysiology of obesity-associated kidney damage (29, 30). Among the proinflammatory adipokines, TNF-α stands out as one of the key mediators of inflammation in adipose tissue and renal dysfunction. Overexpression of TNF-α has frequently been associated with increased production of MCP-1, a chemokine secreted by both adipocytes and macrophages, which has been implicated in adipose tissue expansion. Studies in diet-induced obese animal models have demonstrated elevated levels of cytokines such as IL-6, TNF-α, IL-1, and MCP-1 in renal tissue, correlating with increased interstitial fibrosis and glomerular sclerotic lesions (30, 31). Notably, TNF-α inhibition or deficiency in these models confers protection against obesity-induced albuminuria and structural renal deterioration (10).

Finally, it has been concluded in various CKD models that inflammation and immune system activation share common pathophysiological mechanisms regardless of the underlying etiology, including obesity. Several experimental studies have demonstrated that, in response to pathological stimuli targeting podocytes, there is an upregulation of proinflammatory gene expression, including IL-6, MCP-1, cyclooxygenase-2 (COX-2), and TNF-α; in addition, activation of the nuclear factor kappa B (NF-κB) signaling pathway—a key regulator of inflammation—has been identified. The overexpression of these molecules, particularly in the context of lipid overload as seen in individuals with obesity, promotes a sustained inflammatory response in renal cells, contributing to the maintenance and progression of glomerular damage (24).

## 5 Nephrotoxicity and lipid signaling pathways

In individuals with obesity, chronic energy excess promotes a microenvironment characterized by metabolic stress and persistent inflammation, leading to adipose tissue expansion until adipocytes reach their maximal growth capacity (20). At that point, the excess of toxic lipid species accumulates ectopically in various organs, inducing a harmful effect known as lipotoxicity (32, 33).

In this context, lipotoxicity refers to a condition in which the harmful accumulation of lipids leads to organelle dysfunction, cellular injury, or cell death. Among the deleterious lipids that can potentially accumulate are triglycerides, free fatty acids (FFAs), cholesterol, lysophosphatidylcholine, and ceramides (26, 34). However, the precise mechanisms through which lipids exert adverse effects on the kidney remain unclear (26, 35–37). Lipotoxicity contributes to the intracellular accumulation of toxic lipid intermediates in non-adipose tissues, resulting in cellular dysfunction and, potentially, cell death (lipoapoptosis) (31). Organs most commonly affected include the kidney, liver, heart, pancreas, and skeletal muscle; involvement of these tissues contributes to the development of chronic diseases such as chronic kidney disease (CKD), heart failure, and diabetes mellitus (38).

Under normal circumstances, *de novo* lipid synthesis is very low in renal cells, but ectopic lipid accumulation in the kidney is increasingly common in the obese population. The molecular basis for renal lipid accumulation is poorly understood. The involvement of FXR, SREBP-1c, and PPARα in lipid biosynthesis in the kidney has been identified. Recently, the role of ATP-citrate lyase (ACL), an enzyme that converts citrate to acetyl-CoA, has been identified, demonstrating its role *in vivo* in ectopic renal lipid accumulation and the subsequent renal injury associated with obesity and T2DM (39).

**Figure 2:**
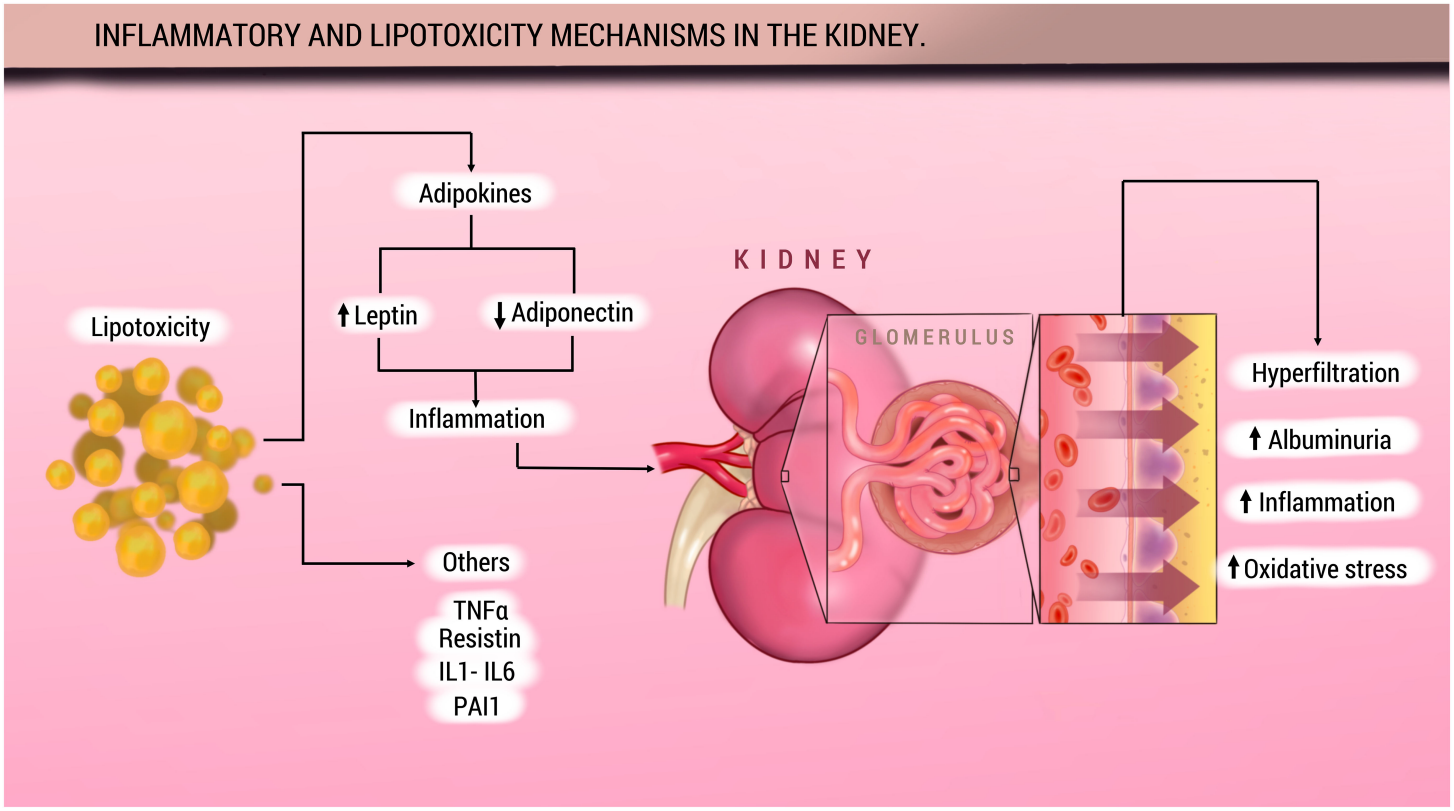
Inflammatory and lipotoxicity in the kidney.

Excessive fat intake contributes to the progression of metabolic diseases through cellular damage and inflammation, processes that promote lipotoxicity (40, 41). Several studies have highlighted the role of lysosomal dysfunction and autophagy in the development of lipotoxicity. In mouse models, a high-fat diet has been shown to cause phospholipid accumulation within lysosomes of renal proximal tubular cells (40). Moreover, this elevated fat intake in obese mice has been associated with stimulation of autophagy, a phenomenon not observed in non-obese mice. Recently, other elements involved in the stimulation of these pathophysiological pathways leading to lipotoxicity have been explored, such as the role of FGF21 (fibroblast growth factor 21), a hormone-like member of the FGF family, which has been linked to impaired renal function (42) **Figure 2**.

Adapted from: Rico-Fontalvo J, et al. (11). Adipose tissue is an important source of production of various active protein factors, known as adipocytokines, which are involved in various metabolic processes. Alterations in the secretion and signaling of adipose-derived molecules during obesity may largely mediate the pathogenesis of metabolic disorders. Adiponectin is a protein secreted primarily by WAT adipocytes. Adiponectin’s main biological functions include increased fatty acid biosynthesis and inhibition of hepatic gluconeogenesis. In patients with CKD, regardless of the presence of obesity, it is associated with elevated serum leptin levels. On the other hand, in obese patients, adiponectin production is decreased, which is why it is believed that it may have a protective effect on the kidney. The imbalance between these adiponectins promotes inflammation. Furthermore, CKD patients show elevated levels of C-reactive protein (CRP), IL-6, and TNF-α. The effects on the glomerulus include hyperfiltration, albuminuria, and oxidative stress. TNF-α: Tumor necrosis factor alpha;IL-1: Interleukin 1; IL-6: Interleukin 6; PAI1: Plasminogen activator inhibitor 1.

Perirenal adipose tissue (PF), which externally surrounds the kidneys, is recognized as a metabolically active fat compartment. This fat depot not only serves as an energy reservoir but also performs relevant endocrine and paracrine functions in the regulation of glucose and lipid homeostasis, as well as in the modulation of inflammatory processes through the synthesis and secretion of various adipokines (15, 43).

Recent evidence has demonstrated an association between PF thickness and an increased risk of developing chronic kidney disease (CKD), suggesting that its quantification could serve as a useful prognostic marker for predicting decreased glomerular filtration rate (GFR) and the presence of proteinuria in individuals with overweight or obesity (15, 44).

Excessive accumulation of perirenal fat (PF) can exert direct mechanical effects on the renal parenchyma and vasculature, generating compression that increases interstitial hydrostatic pressure as this tissue expands, particularly in patients with obesity (45). This condition promotes activation of the renin–angiotensin–aldosterone system (RAAS), as well as increased glomerular filtration and tubular sodium reabsorption—events that contribute to the accelerated progression of kidney disease and the progressive decline in glomerular filtration rate (GFR) (46).

Additionally, PF functions as an active secretory organ, releasing pro-inflammatory mediators and adipokines both paracrinally and systemically. The release of adipokines such as leptin and adiponectin, and cytokines including TNF-α and IL-6, can alter renal hemodynamics and impair the integrity of the vascular endothelium, promoting pathological glomerular hyperfiltration and increased urinary albumin excretion—two key pathophysiological changes in Ob-CKD (43).

First, adiponectin levels are decreased in individuals with obesity, which reduces its anti-inflammatory effects. Several observational clinical studies have demonstrated an independent inverse association between adiponectin levels and albuminuria; obese patients with low adiponectin levels excrete significantly more urinary albumin than obese patients with high adiponectin levels (47).

In contrast, leptin levels are elevated in patients with chronic kidney disease (CKD); this adipokine promotes increased oxidative stress and endothelial dysfunction, contributing to CKD progression. Several studies have shown that serum leptin levels are significantly higher in individuals with obesity compared to those with normal weight (48). This hyperleptinemic state has been implicated in the pathogenesis of renal injury by inducing profibrotic effects and contributing to the development of glomerulosclerosis through mesangial cell activation (16, 32). Furthermore, leptin levels have been observed to progressively increase as renal function declines, suggesting a bidirectional relationship between renal dysfunction and leptin accumulation (16). In advanced stages of CKD, this accumulation may contribute to the development of cachexia, likely through catabolic mechanisms mediated by this adipokine.

Finally, lipotoxicity induces insulin resistance (IR), which directly impacts the loss of renal function. Insulin exerts effects on multiple renal cell types, including mesangial cells, podocytes, and tubular epithelial cells (16). In the context of obesity and IR, there is dysregulated activation of the sympathetic nervous system (SNS) and the renin–angiotensin–aldosterone system (RAAS), along with greater involvement of adipokines, oxidative stress, and proinflammatory pathways—all implicated in the progression of renal dysfunction (16, 46). These mechanisms contribute to endothelial dysfunction, characterized by reduced nitric oxide production, overexpression of angiotensin II type 1 receptors in mesangial cells, and increased synthesis of endothelin-1 and various growth factors, which exacerbate both structural and functional damage to the renal parenchyma (16).

## 6 Clinical perspectives

The understanding that obesity is a proinflammatory and lipotoxic disease that drives cardiovascular-renal-metabolic syndrome requires a paradigm shift in clinical practice. The approach must shift from reactive management of comorbidities to a proactive strategy focused on risk stratification and early intervention on the mechanisms of damage.

### 6.1 Diagnostic and risk strategies

Clinical management should go beyond body mass index (BMI) and serum creatinine to identify patients at higher risk of progression.

- Comprehensive metabolic assessment: Since not all individuals with obesity develop CKD or HF, it is crucial to identify those with “adiposopathy” or an unhealthy metabolic profile. This involves actively assessing for the presence of insulin resistance (e.g., HOMA-IR), dyslipidemia, and inflammatory markers such as high-sensitivity C-reactive protein (hs-CRP), which reflect the systemic proinflammatory state induced by dysfunctional adipose tissue.

- Early detection of kidney damage: Hyperfiltration is one of the first functional alterations in obesity-associated CKD (Ob-CKD Type 1). Therefore, monitoring estimated GFR is essential, but it should be complemented by actively screening for albuminuria.

- Cardiovascular Risk Assessment: In patients with obesity, the high burden of CKD, hypertension, and heart failure should be recognized, and relevant complementary studies should be ordered.

### 6.2 Therapeutic implications

Treatment should focus on modulating central pathophysiological pathways: chronic inflammation and lipotoxicity. The most effective intervention is the reduction of dysfunctional adipose tissue. Weight loss, whether through lifestyle changes, pharmacological treatment, or bariatric surgery, is essential to reduce systemic lipotoxicity, reduce the secretion of proinflammatory adipokines, and improve insulin sensitivity.

Use of drugs with pleiotropic effects: The selection of pharmacological management for comorbidities should prioritize agents with activity on lipotoxicity and inflammation pathways.

- GLP-1 receptor analogs (GLP-1ra): Drugs such as semaglutide or tirzepatide not only induce significant weight loss but have also been shown to improve cardiac structure and function in patients with HFpEF and obesity, likely by reducing inflammation and lipid overload in cardiomyocytes (49–52).

- Sodium-glucose cotransporter 2 inhibitors (SGLT2i): These agents have demonstrated robust cardiorenal benefits. Its mechanisms of action go beyond glycemic control, promoting a metabolic shift that reduces dependence on fatty acid oxidation, thus mitigating cardiac and renal lipotoxicity (53, 54).

- Blockade of the Renin-Angiotensin-Aldosterone System (RAAS): Hyperactivation of the RAAS is a cornerstone of the pathophysiology of both kidney and heart damage in obesity. The use of angiotensin-converting enzyme inhibitors (ACEIs) or angiotensin II receptor blockers (ARBs) is crucial not only for controlling hypertension but also for reducing glomerular hyperfiltration, proteinuria, and fibrosis in both organs.

## 7 Conclusions

Lipotoxicity is a central pathophysiological mechanism in the progression of chronic kidney disease (CKD), particularly in the context of obesity and metabolic syndromes. The accumulation of excess circulating lipids and their ectopic deposition in renal tissue trigger structural and functional injury through multiple pathways, including oxidative stress, mitochondrial dysfunction, chronic inflammation, and activation of profibrotic signaling cascades. These processes directly impair key renal cell populations—such as podocytes, mesangial cells, and proximal tubular epithelial cells—ultimately contributing to progressive nephron loss. A comprehensive understanding of renal lipotoxicity provides a foundation for novel therapeutic strategies aimed at preventing or attenuating kidney injury associated with metabolic derangements, positioning lipid modulation as a promising target in CKD management.
